# RETRACTION: Impact of Moist Wound Dressing on Wound Healing Time: A Meta‐Analysis

**DOI:** 10.1111/iwj.70566

**Published:** 2025-04-18

## Abstract

**RETRACTION:** LiangZ. , LaiP. , ZhangJ. , LaiQ. , and HeL. , “Impact of Moist Wound Dressing on Wound Healing Time: A Meta‐Analysis,” International Wound Journal 20, no. 10 (2023): 4410–4421, 10.1111/iwj.14319.37465989 PMC10681401

The above article, published online on 19 July 2023, in Wiley Online Library (http://onlinelibrary.wiley.com/), has been retracted by agreement between the journal Editor in Chief, Professor Keith Harding; and John Wiley & Sons Ltd. A third party raised concerns about methodological flaws in this article. However, following an investigation by the publisher, all parties have concluded that this article was accepted solely on the basis of a compromised peer review process. The editors have therefore decided to retract the article. The authors did not indicate their agreement with the retraction.

**RETRACTION:** Z. Liang , P. Lai , J. Zhang , Q. Lai , and L. He , “Impact of Moist Wound Dressing on Wound Healing Time: A Meta‐Analysis,” International Wound Journal 20, no. 10 (2023): 4410–4421, 10.1111/iwj.14319.37465989 PMC10681401

The above article, published online on 19 July 2023, in Wiley Online Library (http://onlinelibrary.wiley.com/), has been retracted by agreement between the journal Editor in Chief, Professor Keith Harding; and John Wiley & Sons Ltd. A third party raised concerns about methodological flaws in this article. However, following an investigation by the publisher, all parties have concluded that this article was accepted solely on the basis of a compromised peer review process. The editors have therefore decided to retract the article. The authors did not indicate their agreement with the retraction.

